# An extended TDM method under probabilistic interval-valued hesitant fuzzy environment for stock selection

**DOI:** 10.1371/journal.pone.0252115

**Published:** 2021-05-27

**Authors:** Qasim Noor, Tabasam Rashid, Syed Muhammad Husnine

**Affiliations:** 1 Department of Science and Humanities, National University of Computer and Emerging Sciences, Lahore, Pakistan; 2 Department of Mathematics, School of Sciences, University of Management and Technology, Lahore, Pakistan; University of Defence in Belgrade, SERBIA

## Abstract

Generally, in real decision-making, all the pieces of information are used to find the optimal alternatives. However, in many cases, the decision-makers (DMs) only want “how good/bad a thing can become.” One possibility is to classify the alternatives based on minimum (tail) information instead of using all the data to select the optimal options. By considering the opportunity, we first introduce the value at risk (VaR), which is used in the financial field, and the probabilistic interval-valued hesitant fuzzy set (PIVHFS), which is the generalization of the probabilistic hesitant fuzzy set (PHFS). Second, deemed value at risk (DVaR) and reckoned value at risk (RVaR) are proposed to measure the tail information under the probabilistic interval-valued hesitant fuzzy (PIVHF) environment. We proved that RVaR is more suitable than DVaR to differentiate the PIVHFEs with example. After that, a novel complete group decision-making model with PIVHFS is put forward. This study aims to determine the most appropriate alternative using only tail information under the PIVHF environment. Finally, the proposed methods’ practicality and effectiveness are tested using a stock selection example by selecting the ideal stock for four recently enrolled stocks in China. By using the novel group decision-making model under the environment of PIVHFS, we see that the best stock is *E*_4_ when the distributors focus on the criteria against 10% certainty degree and *E*_1_ is the best against the degree of 20%, 30%, 40% and 50% using the DVaR method. On the other hand when RVaR method is used then the best alternative is *E*_4_ and the worst is *E*_2_ against the different certainty degrees. Furthermore, a comparative analysis with the existing process is presented under the PHF environment to illustrate the effectiveness of the presented approaches.

## 1 Introduction

Every day, everybody makes decisions, and most of them are with some hesitation. For example, what to eat in breakfast, lunch, dinner, time to wake up, choice of clothing to wear, vehicle choice to travel, choice of food type, etc. and most of the decisions do not have a significant impact on life. However, this is not a matter for any firm. Therefore, decision science emerged as a discipline of great interest in both the field of education and the industry. The multi-criteria decision-making (MCDM) helps decide on critical situations. Owing to the complexity and extreme vagueness of practical matters, it is difficult to obtain sufficient and accurate information for real decision-making. Therefore, fuzzy set theory is proposed to present the above noted uncertain information. Numerous decision-making techniques have been emerging nowadays to address real decision-making problems under different fuzzy environments on the basis of different fuzzy sets and then linked to many disciplines [1–3]. Wang et al. [4] investigated the trends and opportunities of the fuzzy set techniques in big data processing and decision making which are two frontier issues in the field. We may find that the general process in these decision-making techniques is the collection of all the fuzzy information used to classify and obtain the best alternative. However, in many decision-making processes, DMs may use limited/partial fuzzy information as a priority, although all data is required. For instance, if DMs want to make a decision based on information about the big gains or huge losses presented by the qualitative fuzzy numbers, they should focus on the tail fuzzy information and collect them to make decisions. The most suitable statement according to the above-cited situation is that DM is trying to know “How worst/better can a thing become”. In the article, we discussed this issue under a fuzzy environment by introducing the VaR and developing the fuzzy VaRs. Moreover, a new tail group decision-making model with PIVHFS is put forward to find the optimal alternative for extreme loss/gain. Hence, the above questions can be answered in this fuzzy decision-making process.

## 2 Literature review

Since Zadeh [5] introduced fuzzy sets in 1965, many extended forms of fuzzy sets have been proposed to provide the DMs with more flexible techniques for getting more rational decision-making results. Torra [6] introduced a fuzzy set by the name of hesitant fuzzy set (HFS), which allowed DMs to insert multiple options on a particular occasion. This theory offers elasticity to DMs, generalizing to the classical fuzzy set concept. Following this, many researchers presented fascinating research interconnected to HFS [7–11]. Numerous scholars have also made new additions to the HFS, which have helped DMs to understand and better model ambiguity and uncertainty. Some of the trendy extensions are triangular HFS [12], generalized HFS [13], interval-valued HFS [14], dual HFS [15], intuitionistic HFS [16], Pythagorean HFS [17], wiggly HFS [18], etc. Inspired by the power of HFS, many operational rules [19] and aggregation operators are also introduced [20–23]. Although HFS is useful and handles vagueness to some extent, the possibility of each element in HFS is the same, which is impossible in real life and creates irrational decision-making results. So HFS cannot handle the situation in which elements have different possibility degrees. To better understand, we take the following examples: Assume that a DM uses HFE, {0.3, 0.5, 0.6}, to identify a risk factor for the investment. The DM can ensure that the risk factor associated with the value of 0.5 is minimal while the risk factor associated with the value of 0.3 is greater than the value of 0.6. Thus, the HFE {0.3, 0.5, 0.6} cannot be used to describe the scene fully. Now consider one more example, assume that the first DM assigns the membership value 0.1 and 0.2 and the second DM assign 0.2 and 0.3 for any event. In such a situation, the HFE {0.1, 0.2, 0.3} is not suitable due to the potential loss of information. Therefore, hesitant fuzzy element (HFE) primarily consists of several membership degrees, but the importance of these membership values is lacking. So HFS cannot handle the situation in which elements have different possibility degrees.

To meliorate the issue, Xu and Zhou [24] comes with a new concept called PHFS, which can handle the set having element containing different possibilities degrees. Encouraged by the influence of PHFS to manage uncertainty and assign a probability value to each HFE, Li and Wang [25] introduced new methods for MCDM, operational laws, and aggregation operators for the PHFS. Hao et al. [26] presented probabilistic dual HFS, which is the modified form of PHFS and used for risk analysis. Gao et al. [27] established a structure by extending a dynamic reference point under the PHFS situation for emergency decision-making. The studies show that PHFS is a powerful extension of HFS and attracted many researchers. However, due to the increasing complexity and uncertainty of the perception in decision-making, in most cases, there were many difficulties for DMs to assess their preferences through crisp values accurately. To get the better of Song et al. [28] proposed a concept of interval-valued PHFS (IVPHFS). The inspiring feature of the IVPHFS is that it could depict two different attributes of a target in a single framework: possible HFS and its corresponding probability interval. Wu et al. [29] defined a PIVHFS as an extended mathematical expression of fuzzy sets and presented the new measure models deduced by the axiomatic concepts of PIVHFSs. Zhai et al. [30] defined a probabilistic interval-valued intuitionistic HFS, which is the generalization of PHFS. As the generalization for the sets interval-valued fuzzy sets and interval-valued HFS (IVHFS), set-valued fuzzy sets and many more were seen in the literature [31, 32].

**Fig 1:**
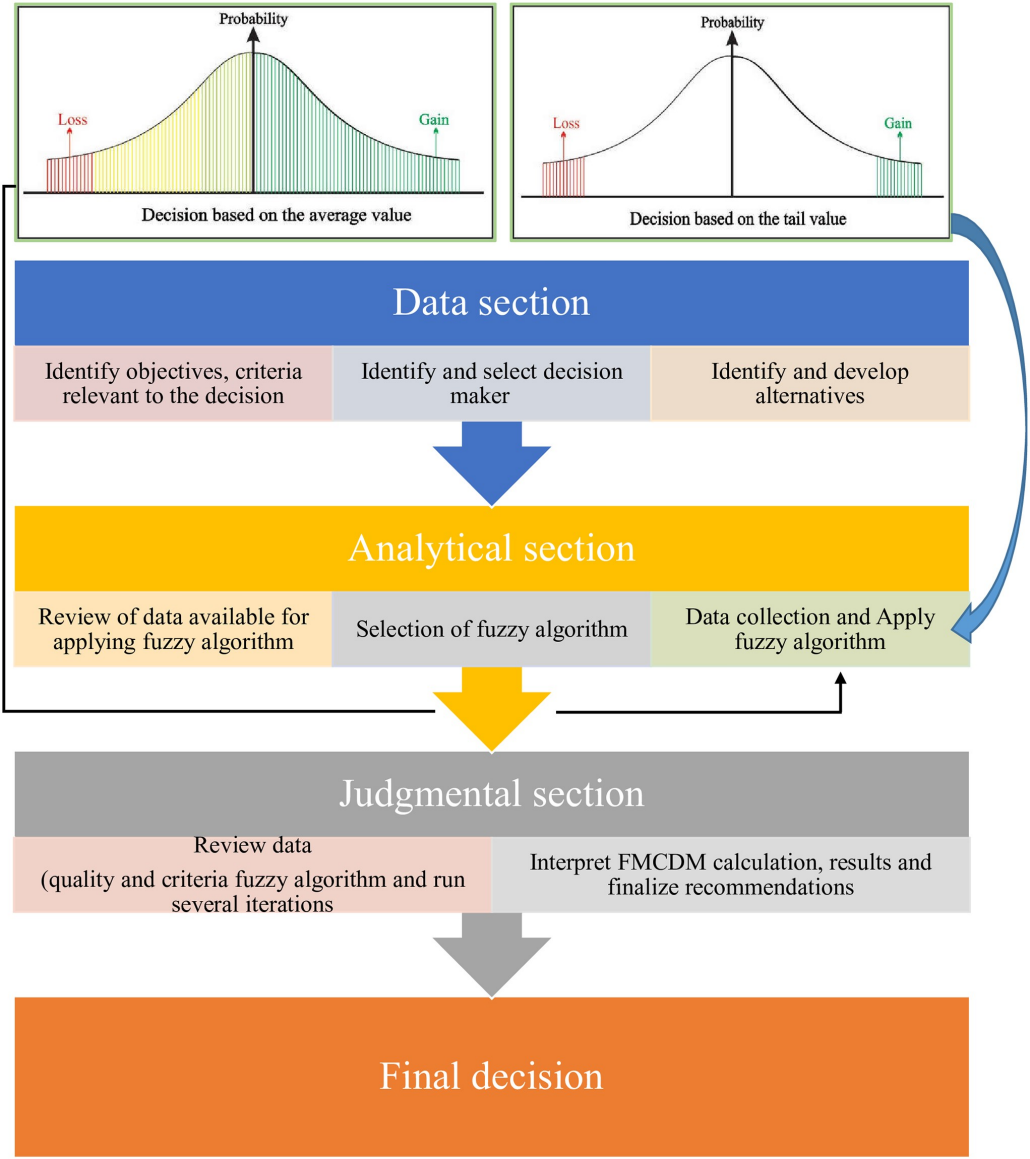
Comparison of TDM and MCDM.

Many fuzzy decision-making techniques based on different fuzzy sets like ELECTRE [33], TOPSIS [34], WASPAS [1] and VIKOR [35] etc. have been introduced to address real decision making under different fuzzy environments. Generally, all the techniques worked somehow on the same pattern and can be seen in Fig 1, representing the fuzzy multi-criteria decision making (FMCDM) process. Zhou and Xu [36] explored the technique called tail decision making (TDM) under PHF environment. There are many factors involved in the decision-making process. However, some of the elements are given in Fig 1 to quickly understand the difference between the traditional methods (TOPSIS, VIKOR, etc.) used in decision-making and TDM.

In the process of all fuzzy decision-making techniques, DMs aggregated all the pieces of information and obtained the best alternatives. However, in many real-world problems, while fuzzy decision-making processes, to make a decision, DMs only use partial or primary information as a priority. For example, if DM wanted to decide the extreme gain/loss using qualitative (fuzzy elements) information, the DM’s main focus is on the tail information. Using the tail information, DM has to decide and wish to know the formal statement as “how worst a thing can be or how worthy a thing can be?”. The term VaR was used in the literature to describe this type of situation. Fuzzy data is used to introduce fuzzy VaRs and as a basic tool to construct TDM under a PIVHF environment. VaR is a risk analysis measurement tool used in the financial field to measure the risk of loss for investments. It estimated the set of investments that might result in loss with a given probability *p*, under normal market conditions. Moreover, for a given collection and the probability *p*, VaR is a threshold value showing that the collection’s loss did not exceed the value [37]. Using the definition of VaR, different VaRs can be seen in the literature [38–42]. All the VaR discussed in the literature used the quantitative data in such a way that VaR is not suitable under a general fuzzy environment. Zhou and Xu [36] introduced fuzzy VaR, which could handle the qualitative information under the environment of PHFS.

The literature revealed that whenever a new set or new technique is introduced, the general rule is presented to handle the problem in a certain and accurate way. However, there is no research on the TDM under the PIVHF environment at present. So, taking inspiration from Zhou and Xu [36] work using hesitant value at risk (HVaR) and expected hesitant VaR (EVaR) under probabilistic hesitant fuzzy environment techniques, this novel presented the generalization of the idea named as, deemed VaR (DVaR) and Reckoned VaR (RVaR) under PIVHF environment. The extension provided more authentic and certain decisions as compared to the results obtained from HVaR and EVaR.

The rest of the paper is organized as follows: Section 3 provides the fundamental concepts used in TDM. Section 4 presents an extended form of VaR measurement under the PIVHF environment; namely, the DVaR is proposed and deduces its comparison rule. Furthermore, another improved measurement tool RVaR is developed to handle every type of information in TDM under the PIVHF environment. TDM process is discussed in Section 5 under the PIVHF environment. Section 6 employs an example of selecting the ideal stock, keeping in view the extreme risk/loss. Section 7 concludes the study and elaborates on future studies.

## 3 Preliminaries

In the current section, the PIVHFS and the PIVHFE are introduced. Then, their operational rules and laws are provided, which are used to calculate the TDM under the PIVHF environment.

**Definition 3.0.1**. [6] *Let X be any universe of discourse. The hesitant fuzzy set(HFS) H on X is a function h*_*H*_ (*x*): *X* → [0, 1] *when applied to X returns a finite subset of* [0, 1].

Mathematically, Xia and Xu [43] defined it as:

$$\begin{array}{c} H=\{\langle x,h_{H}(x):x\in X\rangle \}, \end{array}$$

where *h*_*H*_ (*x*) is the discrete set of values from [0, 1] also called hesitant fuzzy element.

Practically, many decision-making problems are challenging to be represented by accurate values. Thus, interval-valued data will be preferred. To illustrate the uncertainty in the decision process more efficiently, IVHFS was defined by Chen [31] as:

**Definition 3.0.2**. *Let X be any given set, and C* [0, 1] *be the set of all closed sub-interval of* [0, 1]. *IVHFS on X is defined as*

$$\begin{array}{c} \tilde{H}=\{\langle x_{i},{\tilde{h}}_{\tilde{H}}(x_{i})\rangle :x_{i}\in X\}, \end{array}$$

where ${\tilde{h}}_{\tilde{H}}(x_{i}):X\to C[0,1]$ denotes all possible interval-valued membership degree of the element *x*_*i*_ ∈ *X*. For convenience, we call

$$\begin{array}{c} \;{\tilde{h}}_{\tilde{H}}(x_{i})=\{\gamma :\gamma \in {\tilde{h}}_{\tilde{H}}(x_{i})\} \end{array}$$

interval-valued hesitant fuzzy element. Here *γ* = [*γ*^−^, *γ*^+^] is an interval number, *γ*^−^ and *γ*^+^ represent the lower and upper limits of *γ*.

It is noted that the preferences usually based on HFS, or IVHFS are not homogeneous according to many DMs, which led to a problem for preferences [36, 44, 45]. Therefore, to enhance the preferences in decision-making problems, Zhu and Xu [46] extended the HFS to PHFS, defined as:

**Definition 3.0.3**. *Let X be any universe of discourse having n elements, PHFS can be expressed by an expression*

$$\begin{array}{c} H_{p}=\{\langle x,h_{H_{p}}(x):x\in X\rangle \}, \end{array}$$

where $h_{H_{p}}(x)\in [0,1],$ represent the membership degree of the element *x* ∈ *X*. For the simplicity, we call

$$\begin{array}{c} h_{H_{p}}(x)=h(p)=\{\gamma_{i}(p_{i})|i=1,2,3,\ldots ,|h(p)|\}, \end{array}$$

where *p*_*i*_ is the probability of the belonging degree *γ*_*i*_ of PHFE, |*h*(*p*)| is the number of all belonging degrees and $\sum_{i=1}^{\left|h(p)\right|}p_{i}=1$.

As mentioned above that in many decision-making problems, data is not accurate. Thus, intervals instead of a single hesitant value will be preferred. Similarly to the case with PHFSs, to overcome the situation, Wu et al. [29] defined the PIVHFS as below:

**Definition 3.0.4**. *Let X be a universal set, and C* [0, 1] *be the set of all closed sub-intervals of* [0, 1]. *A PIVHFS on X is*

$$\begin{array}{c} \tilde{H}=\{\langle x_{i},{\tilde{h}}_{\tilde{H}}(x_{i})\rangle |x_{i}\in X,i=1,2,3,\ldots ,n\}, \end{array}$$

where

$$\begin{array}{c} {\tilde{h}}_{\tilde{H}}(x_{i})=\{([\gamma_{k}^{-},\gamma_{k}^{+}],p_{\gamma_{k}})|[\gamma_{k}^{-},\gamma_{k}^{+}]\in {\tilde{h}}_{\tilde{H}}(x_{i}),p_{\gamma_{k}}\in [0,1],\sum_{k=1}^{l_{\tilde{h}}}p_{\gamma_{k}}=1\} \end{array}$$

*denotes all possible interval-valued membership degree of the element x*_*i*_ ∈ *X*. *For simplicity, a PIVHFE, will be denoted by* ${\tilde{h}}_{\tilde{H}}(x_{i})=\tilde{h}$

The basic operational laws of PIVHFEs and the assessment rules are defined as follows:

**Definition 3.0.5**. [29] *The score function of the PIVHFE can be calculated by the expression*

$$\begin{array}{c} s(\tilde{h})=\frac{1}{l_{\tilde{h}}}\sum_{k=1}^{l_{\tilde{h}}}(\gamma_{k}^{-}+\gamma_{k}^{+})p_{\gamma_{k}}, \end{array}$$ (1)

where $l_{\tilde{h}}$ is the number of intervals in $\tilde{h},$ and the value of $s(\tilde{h})$ is belong to [0, 1]. The deviation function of the PIVHFE is defined as:

$$\begin{array}{c} d(\tilde{h})=\sum_{k=1}^{l_{\tilde{h}}}[0.5(\gamma_{k}^{-}+\gamma_{k}^{+})-s(\tilde{h}){]}^{2}.p_{\gamma_{k}} \end{array}$$ (2)

The comparison rules of two PIVHFEs ${\tilde{h}}_{1}$ and ${\tilde{h}}_{2}$ can be stated as follows:

1. If $s({\tilde{h}}_{1})>s({\tilde{h}}_{2}),$ then ${\tilde{h}}_{1}≻{\tilde{h}}_{2}$, which means that ${\tilde{h}}_{1}$ is preferable to ${\tilde{h}}_{2}.$
2. If $s({\tilde{h}}_{1})=s({\tilde{h}}_{2})$, then
  (a) If $d({\tilde{h}}_{1})>d({\tilde{h}}_{2})$, then ${\tilde{h}}_{1}≺{\tilde{h}}_{2}$, which means that ${\tilde{h}}_{2}$ is preferable to ${\tilde{h}}_{1}.$
  (b) If $d({\tilde{h}}_{1})<d({\tilde{h}}_{2})$, then ${\tilde{h}}_{1}≻{\tilde{h}}_{2}$, which means that ${\tilde{h}}_{1}$ is preferable to ${\tilde{h}}_{2}.$
  (c) If $d({\tilde{h}}_{1})=d({\tilde{h}}_{2})$, then ${\tilde{h}}_{1}∼{\tilde{h}}_{2}$, which means that ${\tilde{h}}_{1}$ and ${\tilde{h}}_{2}$ has the same preference.

The symbols “>” and “<” are different from “≻” and “≺” The symbols “≺” and “≻” are employed to relate the two PIVHFE. While the symbols “>” and “<” gives us the comparison of two real numbers.

**Definition 3.0.6**. [29] *Let* $\tilde{h}=\underset{\gamma \in \tilde{h}}{\cup }\{([\gamma^{-},\gamma^{+}],p)\},\tilde{h_{1}}=\underset{\gamma_{1}\in \tilde{h}}{\cup }\{([\gamma_{1}^{-},\gamma_{1}^{+}],p_{1})\}$ *and* ${\tilde{h}}_{2}=\underset{\gamma_{{}_{2}}\in \tilde{h}}{\cup }\{([\gamma_{2}^{-},\gamma_{2}^{+}],p_{2})\}$ *be the three PIVHFEs, then the basic algebra on PIVHFE are defined as*:

1. $\left(\tilde{h}{)}^{c}=\underset{\gamma \in \tilde{h}}{\cup }\{([1-\gamma^{+},1-\gamma^{-}],p)\right\}$
2. ${\tilde{h}}_{1}⊕{\tilde{h}}_{2}=\underset{\gamma_{1}\in {\tilde{h}}_{1},\gamma_{2}\in {\tilde{h}}_{2}}{\cup }\{([\gamma_{1}^{-}+\gamma_{2}^{-}-\gamma_{1}^{-}.\gamma_{2}^{-},\gamma_{1}^{+}+\gamma_{2}^{+}-\gamma_{1}^{+}.\gamma_{2}^{+}],p_{1}p_{2})\}$
3. ${\tilde{h}}_{1}⊗{\tilde{h}}_{2}=\underset{\gamma_{1}\in {\tilde{h}}_{1},\gamma_{2}\in {\tilde{h}}_{2}}{\cup }\{([\gamma_{1}^{-}.\gamma_{2}^{-},\gamma_{1}^{+}.\gamma_{2}^{+}],p_{1}p_{2})\}$
4. $\lambda (\tilde{h})=\underset{\gamma \in \tilde{h}}{\cup }\{([1-(1-\gamma^{-}{)}^{\lambda },1-(1-\gamma^{+}{)}^{\lambda }],p)\}$, where λ > 0
5. $\left(\tilde{h}{)}^{\lambda }=\underset{\gamma \in \tilde{h}}{\cup }\{([(\gamma^{-}{)}^{\lambda },(\gamma^{+}{)}^{\lambda }],p)\right\}$, where λ > 0

To facilitate to compare the magnitude of PIVHFEs, we give the properties of probabilistic interval numbers.

**Definition 3.0.7**. *Let A* = ([*a*_1_, *a*_2_], *p*_1_) *and B* = ([*b*_1_, *b*_2_], *p*_2_) *be two probabilistic interval number, and* λ ≥ 0, *then*

1. *A* < *B* if $\frac{a_{1}+a_{2}}{2}<\frac{b_{1}+b_{2}}{2}$
2. *A* < *B* if $\frac{a_{1}+a_{2}}{2}=\frac{b_{1}+b_{2}}{2}$ and *p*_1_ < *p*_2_
3. *A* = *B* if $\frac{a_{1}+a_{2}}{2}=\frac{b_{1}+b_{2}}{2}$ and *p*_1_ = *p*_2_

Inspired by the concept of Zhou et al. [36] HVaR and EVaR for TDM under PHF environment, the extension under PIVHF environment is presented in the next section.

## 4 Two VaR measurements of the PIVHFE

In many group decision-making problems, data is sometimes not presented by accurate values; thus, data is preferred in intervals. Because, of the increasing complexity and uncertainty of the perception in decision-making, in most cases, there were many difficulties for DMs to accurately assess their preferences through crisp values specified but can be expressed with an interval value. Zhou and Xu [36] gave two concepts, HVaR and EVaR for calculating VaR under PHF environment. However, due to uncertainty, suppose DMs face difficulties to accurately provide the membership values in crisp values, so they have the option to provide values in the form of intervals. Therefore, alternatives may not be certain. Instead of using a single hesitant value, interval hesitant values are used and proposed two ideas DVaR and RVaR, which provided the more certain and accurate alternatives.

The score values of any fuzzy numbers are actually the weighted average value of the whole data and used to make decisions for the different fuzzy numbers. A bigger score value means the better would be the result. Practically, it looks like a reasonable method. Let us consider a situation that, a corporation/company having *n* number of stockholders/distributors wants to purchase a newly established bank (A, B, C, D). After the market survey, stockholders observe that the banks are currently on a loss but have a strong probability that they will soon become on the profitable stage. The company has to buy one bank, and he has to make such a choice where chances of loss are minimized. This seems that the company will take a risk and focus on the question “how much the worst stock can be.” or simply what is the maximum loss he has to bear if the company buys any one of the banks. In this situation, we do not need the weighted value of the whole data but focuses on the result that what is the maximum gain/loss. As banks are newly established, so more chances are lacking quantitative data, then the TDM approach with the PIVHF information should be introduced.

A review of the literature reveals that interval-valued data gives one possibility to get certain and authentic alternatives compared to insufficiency in available information; it may be difficult for DMs to quantify their opinions with a crisp number. Therefore, data can be represented by an interval number within [0, 1]. But the main problem in interval-valued data is the ordering. Since the number of interval values for different PIVHFEs could be different, and the interval values are usually out of order and are hard to deal with intervals instead of single values because in numbers ordering are much easy, we can easily compare that which values are less and which one is greater. Several orders have been studied and defined in the literature between interval-valued fuzzy sets [47].

### 4.1 Deemed VaR of the PIVHFE

**Fig 2:**
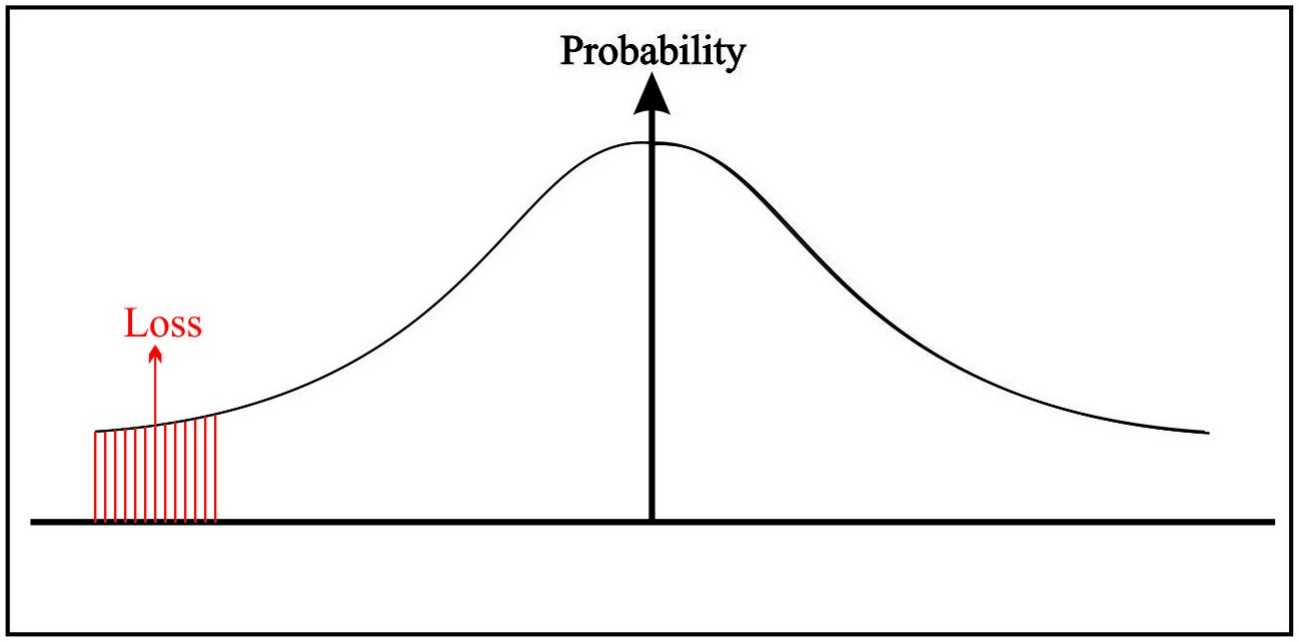
TDM based on the loss tail under PIVHF environment.

**Fig 3:**
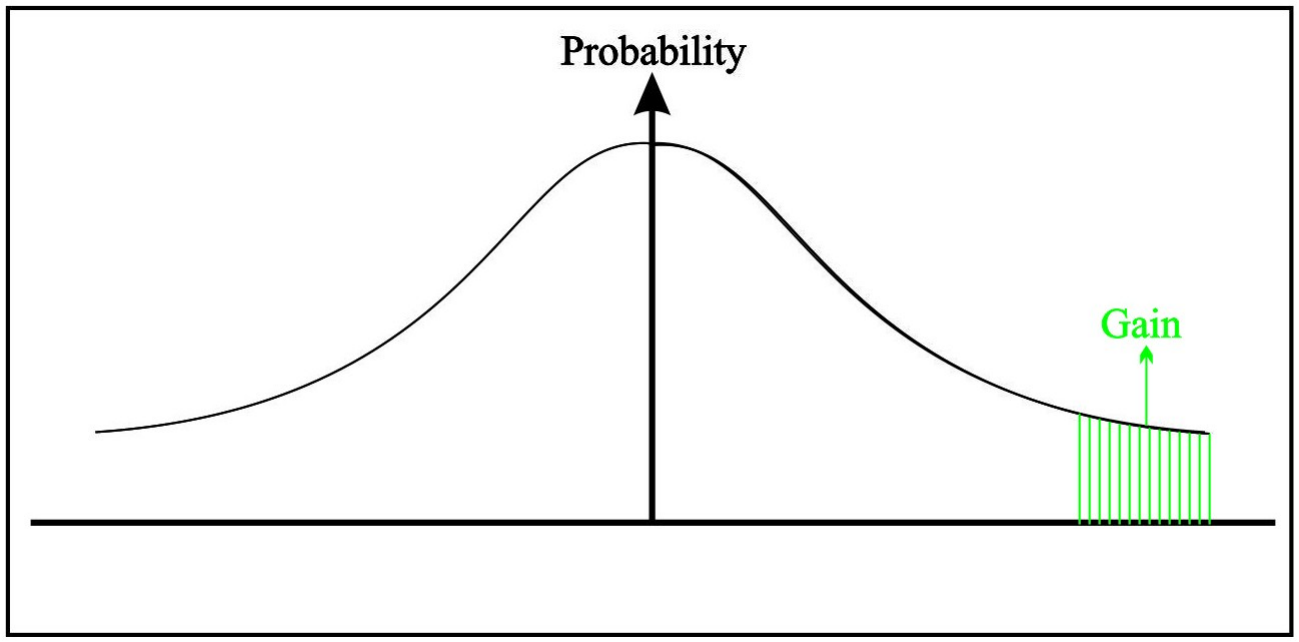
TDM based on the gain tail under PIVHF environment.

Basically, the tail decision’s information is of two types; namely, the left tail (loss tail) and right tail (profit tail) can be seen in Figs 2 and 3, respectively. The conclusions based on the two tails are of a different type; the left tail gives us the loss information (how bad a thing can be), and the right tail gives us the profit information (how good a thing can be). The process to find the best/worst alternatives for profit/loss values is the same. In the paper, we proposed the VaR under the PIVHF environment based on the left tail. On the other hand, VaR for the right tail can be calculated in the same manner.

VaR of the PIVHFE, which is going to be used to find the optimal alternative for tail information under PIVHF information, is defined as:

**Definition 4.1.1** *Let* $\tilde{h}=\{([\gamma_{1}^{-},\gamma_{1}^{+}],p_{\gamma_{1}}),([\gamma_{2}^{-},\gamma_{2}^{+}],p_{\gamma 2}),\ldots ([\gamma_{n}^{-},\gamma_{n}^{+}],p_{\gamma n})\}$ *be a PIVHFE, where* $\gamma_{i}^{+},\gamma_{i}^{-},p_{\gamma_{i}}\in [0,1]$, (*i* = 1, 2, 3, …, *n*), $\sum_{i=1}^{n}p_{\gamma_{i}}=1$ *and p*_0_ = 0 *is a known parameter. If we are X percent sure that the element* $[\gamma_{i}^{-},\gamma_{i}^{+}]|p_{\gamma_{i}},$, (*i* = 1, 2, …, *n*) *is not less than D, then D is called DVaR of the PIVHFE* $\tilde{h}$, *with the degree of certainty X. Mathematically it can be represented as*:

$$\begin{array}{c} DVaR(\tilde{h})=D=g^{-1}\{p_{\gamma_{i}}|\sum_{i=0}^{m-1}p_{\gamma_{i}}<X\leq \sum_{i=0}^{m}p_{\gamma_{i}},(i=1,2,\ldots ,n),(1\leq m\leq n)\;\text{and}\;X>0\}, \end{array}$$ (3)

where $g(p_{\gamma_{i}})=(0.5[\gamma_{i}^{-}+\gamma_{i}^{+}])$ is the density function of $\tilde{h}$.

The above expression calculation process is simple and answers to DMs of the question that “how bad/worst can a thing become.” It is the question that DMs want in this type of decision-making.

From the Eq 3, we observe that DVaR can directly be calculated based on the cumulative distribution of the PIVHFE. The calculation rules are defined as:

1. If $\sum_{i=0}^{m-1}p_{\gamma_{i}}<\bar{X}\leq \sum_{i=0}^{m}p_{\gamma_{i}}$, then $\bar{D}=(0.5[\gamma_{m}^{-}+\gamma_{m}^{+}])$, where (*i* = 1, 2, …, *n*), (1 ≤ *m* ≤ *n*) and $\bar{X}>0$
2. If $\sum_{i=0}^{m-1}p_{\gamma_{i}}=\bar{X}$, then $\bar{D}=(0.5[\gamma_{m-1}^{-}+\gamma_{m-1}^{+}])$, where (*i* = 1, 2, …, *n*), (1 ≤ *m* ≤ *n*) and $\bar{X}>0$

For any two PIVHFEs ${\tilde{h}}_{1}$ and ${\tilde{h}}_{2}$, if $DVaR({\tilde{h}}_{1})=D_{1}$ and $DVaR({\tilde{h}}_{2})=D_{2}$, then we have

1. If *D*_1_ > *D*_2_, then $DVaR({\tilde{h}}_{1})≻DVaR({\tilde{h}}_{2})$.
2. If *D*_1_ < *D*_2_, then $DVaR({\tilde{h}}_{1})≺DVaR({\tilde{h}}_{2})$.
3. If *D*_1_ = *D*_2_, then $DVaR({\tilde{h}}_{1})∼DVaR({\tilde{h}}_{2})$.

In the subsection, DVaR of the PIVHFE, its computation method and comparison rules are provided.

### 4.2 Reckoned VaR of the PIVHFE

In the previous subsection, DVaR under the PIVHF environment is proposed, which can easily be applied to PIVHFE. A problem occurs in the process of DVaR when PIVHFEs share the same interval or midpoint of the interval concerning same occurrence probabilities, as shown in the example:

**Example 1**. *Consider the two PIVHFEs*

$$\begin{array}{c} {\tilde{h}}_{1}=\{([\gamma_{1}^{-},\gamma_{1}^{+}],p_{\gamma_{1}}),([\gamma_{2}^{-},\gamma_{2}^{+}],p_{\gamma 2}),\ldots ,l_{{\tilde{h}}_{1}}\} \end{array}$$

$$\begin{array}{c} {\tilde{h}}_{2}=\{([\gamma_{2}^{-},\gamma_{2}^{+}],p_{\gamma_{1}}+p_{\gamma 2}),([\gamma_{3}^{-},\gamma_{3}^{+}],p_{\gamma 3}),\ldots ,l_{{\tilde{h}}_{2}}\} \end{array}$$

and take $X=p_{\gamma_{1}}+p_{\gamma 2}$, then by Eq 3 we see that

$$\begin{array}{c} DVaR({\tilde{h}}_{1})=DVaR({\tilde{h}}_{2})=(0.5[\gamma_{2}^{-}+\gamma_{2}^{+}]) \end{array}$$

Obviously, the above result will give us the wrong information because ${\tilde{h}}_{1}$ has lesser value than ${\tilde{h}}_{2}$ with certainty degree $p_{\gamma_{1}}+p_{\gamma_{2}}.$ It is observed that the tail information of ${\tilde{h}}_{1}$ is $\left([\gamma_{1}^{-},\gamma_{1}^{+}],p_{\gamma_{1}}\right)$ and $\left([\gamma_{2}^{-},\gamma_{2}^{+}],p_{\gamma_{2}}\right)$ with degree of certainty $p_{\gamma_{1}}+p_{\gamma_{2}}$ and tail information of ${\tilde{h}}_{2}$ is $\left([\gamma_{2}^{-},\gamma_{2}^{+}],p_{\gamma_{2}}\right)$ with degree of certainty $p_{\gamma_{1}}+p_{\gamma_{2}}.$ Thus, the result should be of the kind ${\tilde{h}}_{1}≺{\tilde{h}}_{2}.$ Therefore, DVaR may produce the wrong results.

To deal with such a problem, we propose RVaR, which can measure the better alternative even if two intervals share the same midpoint for TDM under the PIVHF environment.

**Definition 4.2.1**. *Let* $\tilde{h}=\{([\gamma_{1}^{-},\gamma_{1}^{+}],p_{\gamma_{1}}),([\gamma_{2}^{-},\gamma_{2}^{+}],p_{\gamma 2}),\ldots ([\gamma_{n}^{-},\gamma_{n}^{+}],p_{\gamma n})\}$ *be a PIVHFE, where* $\gamma_{i}^{-},\gamma_{i}^{+}\in [0,1]$, $p_{\gamma_{i}}\in [0,1]$ $\sum_{i=1}^{n}p_{\gamma_{i}}=1$ *and p*_0_ = 0 *is a known parameter. If we are X percent sure that the element* $[\gamma_{i}^{-},\gamma_{i}^{+}],p_{\gamma i}$ (*i* = 1, 2, …, *n*) *is not less than R, then R is called RVaR of the PIVHFE* $\tilde{h}$, *with the degree of certainty X*.

Mathematically can be represented as:

$$\begin{array}{c} \text{RVaR}(\tilde{h})=R=\sum_{i=0}^{m-1}f(\gamma_{i})+\gamma_{m}X-\gamma_{m}\sum_{i=0}^{m-1}p_{{}_{\gamma i}}, \end{array}$$ (4)

where $\sum_{i=0}^{m-1}p_{\gamma_{i}}<X\leq \sum_{i=0}^{m}p_{\gamma_{i}},(i=1,2,\ldots ,n),(1\leq m\leq n)$, *X* > 0 and $f(\gamma_{i})=(\gamma_{i}^{-}+\gamma_{i}^{+}).p_{{}_{\gamma i}}$ is the density function of $\tilde{h}$. For simplicity we take the parameters *p*_0_ = 0 and $[\gamma_{0}^{-},\gamma_{0}^{+}]=0$

To facilitate to compare the magnitude of different PIVHFEs, we give the properties. For two PIVHFEs ${\tilde{h}}_{1}$ and ${\tilde{h}}_{2}$, if $\text{RVaR}({\tilde{h}}_{1})=R_{1}$ and $\text{RVaR}({\tilde{h}}_{2})=R_{2}$, then

1. If *R*_1_ > *R*_2_, then ${\tilde{h}}_{1}≻{\tilde{h}}_{2}$.
2. If *R*_1_ < *R*_2_, then ${\tilde{h}}_{2}≻{\tilde{h}}_{1}$.
3. If *R*_1_ = *R*_2_, then ${\tilde{h}}_{1}∼{\tilde{h}}_{2}$.

Furthermore, to easily understand the final results of the above-proposed methods DVaR and RVaR, a simple example is given below:

**Example 2**. *A company wants to purchase a newly established bank (A, B). By survey the report, the company provides the evaluation information for the two banks using the PIVHFEs as*:

$$\begin{array}{c} {\tilde{h}}_{A}=\{([0.1,0.2],0.2),([0.3,0.4],0.5),([0.35,0.45],0.15),([0.45,0.55],0.15)\} \end{array}$$

$$\begin{array}{c} {\tilde{h}}_{B}=\{(([0.05,0.15],0.05),[0.1,0.2],0.15),([0.25,0.35],0.4),([0.4,0.5],0.2),([0.5,0.6],0.2)\} \end{array}$$

What is the optimal alternative?

1. If the company wants a decision using the overall value, then DMs uses the score value of PIVHFE, then
  $$\begin{array}{c} s({\tilde{h}}_{A})=0.17\;\text{and}\;s({\tilde{h}}_{B})=0.139 \end{array}$$
2. If the company emphasizes on the question that “how worst can be the the bank under certainty degree of 20%”, Then by using Eq 3 and the Eq 4, we get the following values:
  $$\begin{array}{c} \text{DVaR}({\tilde{h}}_{A})=0.15\;\text{and}\;\text{DVaR}({\tilde{h}}_{B})=0.15 \end{array}$$
  and
  $$\begin{array}{c} \text{RVaR}({\tilde{h}}_{A})=0.06\;\text{and}\;\text{RVaR}({\tilde{h}}_{B})=0.055 \end{array}$$
  It can be seen that when DVaR method is applied, we get the same values so, ${\tilde{h}}_{A}∼{\tilde{h}}_{B}$. But on the other hand, it is observed, ${\tilde{h}}_{A}≻{\tilde{h}}_{B}$ when RVaR is applied. It clearly shows that RVaR is more suitable than DVaR to differentiate the PIVHFEs. Hence bank A is the best alternative if the company focus on how worst can be the selection among two banks.

From the example, we can see that DVaR is much simple and straightforward than the RVaR. Thus, DVaR is used in TDM with some extreme values or the complicated TDM. In the paper, RVaR is employed for the TGDM using the PIVHF environment. The TDM and TGDM steps are proposed based on RVaR. It was further deduced that both DVaR and RVaR are applied to show the efficiency and modifications in Section 6.

### 4.3 TDM using RVaR

RVaR focuses on the statement that “we are *X* per cent certain that the reckoned value of the result is not less than *R*.” Therefore, RVaR can be used to decide by the DMs who focus on extreme gain/loss. The calculation steps for RVaR can be defined as follows:

1. For TDM under PIVHF environment, there are *n* alternatives, namely *E*_*i*_(*i* = 1, 2, 3, …, *n*) and the information vectors as PIVHFEs
  $$\begin{array}{c} {\tilde{h}}_{i}=\{([\gamma_{i1}^{-},\gamma_{i1}^{+}],p_{\gamma_{i1}}),([\gamma_{i2}^{-},\gamma_{i2}^{+}],p_{\gamma_{i2}}),\ldots ,l_{\tilde{h}i}\} \end{array}$$
2. Define the certainty degree *X* according to the need of the DMs or appetite of risk taken by DMs.
3. When certainty degree decided, then according to certainty degree obtain the tail information of the PIVHFEs after cumulative the overall PIVHFEs ${\tilde{h}}_{i}(i=1,2,3,\ldots ,n)$ with respect to the probabilities.
4. Now calculate the RVaR, *R*_*i*_ for the PIVHFEs ${\tilde{h}}_{i}(i=1,2,3,\ldots ,n)$ using Eq 4.
  (a) If the criterion index under consideration is a benefit, the bigger *R*_*i*_ the best alternative be.
  (b) If the criterion index under consideration is cost, the smaller *R*_*i*_ the best alternative be.

## 5 Group decision making using RVaR

In Section 4, the two decision-making approaches, DVaR and RVaR, are presented under the PIVHF environment. The decision-making process can be carried out by PIVHFE information for one DM. Further, if different DMs provide PIVHFEs, then the idea can also be extended to TGDM.

**Step 1. Collect** *k* **PIVHFE information matrices**

For the TGDM problem under PIVHF environment, let us consider *E* = {*E*_1_, *E*_2_, …, *E*_*n*_} be *n* alternatives and *D* = {*d*_1_, *d*_2_, …, *d*_*k*_} be *k* DMs. The DM *d*_*j*_ provides the information and the probabilities of the alternative *E*_*i*_ under some criteria (benefit) represented by PIVHFE

$$\begin{array}{c} {\tilde{h}}_{ij}=\{([\gamma_{ij1}^{-},\gamma_{ij1}^{+}],p_{\gamma_{ij1}}),([\gamma_{ij2}^{-},\gamma_{ij2}^{+}],p_{\gamma_{ij2}}),\ldots ,l_{\tilde{h}ij}\} \end{array}$$

Then we obtain *k* PIVHFE vectors ${\tilde{H}}_{j}=({\tilde{h}}_{ij}{)}_{n\times 1}(i=1,2,\ldots n;j=1,2,\ldots ,k)$.

**Step 2**. **Calculating the weights of the DMs**

As more DMs are involved so the information provided is different. Further authentic information providers have more weight than DM without authentic information. Higher weights will be assigned to those DMs who will provide accurate information, or in other words, greater weights will be assigned to those who have a smaller deviation degree. In the paper, the deviation degree of PIVHFE represents the accuracy degree. Weights of the DMs can be calculated using the dynamic model defined by Zhou and Xu [36] as follows:

$$\begin{array}{c} \text{min}\;P=\sum_{f=1}^{k-1}\sum_{t=2}^{k}(t_{ft}^{+}+t_{ft}^{-}) \end{array}$$

s.t

$$\begin{array}{c} \begin{array}{c} w_{if}.\sqrt{\sum_{t=1}^{\#{\tilde{h}}_{ig}}[(\gamma_{if}-s({\tilde{h}}_{if}){)}^{2}p_{{}_{\gamma_{if}}}]}-\sqrt{\sum_{t=1}^{\#{\tilde{h}}_{ig}}[(\gamma_{it}-s({\tilde{h}}_{it}){)}^{2}p_{{}_{\gamma_{it}}}]}-t_{ft}^{+}+t_{ft}^{-}=0\\ s({\tilde{h}}_{ij})=\sum_{t=1}^{\#{\tilde{h}}_{if}}\gamma_{ij}p_{\gamma_{ij}}\\ \sum_{j=1}^{k}w_{ij}=1,\;\;w_{ij}\geq 0\\ t_{ft}^{+},t_{ft}^{-}>0\\ f<t,\;\;\;f,t\in \{1,2,\ldots ,k\}\\ i=1,2,\ldots ,n;\;\;\;j=1,2,\ldots ,k \end{array}\} \end{array}$$ (5)

where $t_{ft}^{-}$ is the negative deviation and $t_{ft}^{+}$ is the positive deviation.

By using the Eq 5 we obtain the *n* weight vectors *W*_*i*_ = (*w*_*i*1_, *w*_*i*2_, …, *w*_*ik*_), *i* = 1, 2, …, *n*. Put the vectors in matrix to form a weight matrix *W* = (*w*_*ij*_)_*n*×*k*_, where *i* = 1, 2, …, *n* and *j* = 1, 2, …, *k*.

**Step 3**. **Aggregate the PIVHFE information vectors**

From the information vectors obtained in step 1 ${\tilde{H}}_{j}=({\tilde{h}}_{ij}{)}_{n\times 1}$, we obtain *n* PIVHFE vectors ${\hat{H}}_{i}=({\tilde{h}}_{i1},{\tilde{h}}_{i2},\ldots ,{\tilde{h}}_{ik}),i=1,2,\ldots ,n$. Now by using the weighted operation $W_{i}\times ({\hat{H}}_{i}{)}^{T}$, aggregate *n* PIVHFE information vectors and obtain the vectors ${\tilde{H}}_{i}=({\tilde{h}}_{i}{)}_{n\times 1}$, where ${\tilde{h}}_{i}=W_{i}\times ({\hat{H}}_{i}{)}^{T}$ and *i* = 1, 2, …, *n*. Use definition 3.0.7 arrange the elements of ${\tilde{h}}_{i}(i=1,2,3,\ldots ,n)$ in ascending order so that tail information can be obtained according to the risk appetite of the DMs.

**Step 4**. **Set the certainty degree of the PIVHFE**

After cumulative distribution collect the tail information of the PIVHFEs, set the basic parameters (certainty degree) by keeping in view the affordability of risk taken by DMs.

**Step 5**. **Conclusion by using the RVaR**

By using the Eq 4 calculate the *R*_*i*_ of the PIVHFEs ${\tilde{h}}_{i}(i=1,2,\ldots ,n)$ according to the defined parameters. Final conclusion can be drawn based on the criteria index as follows:

1. If the criteria is benefit, then the bigger *R*_*i*_ the superior alternative be.
2. If the criteria is cost, then the smaller *R*_*i*_ the superior alternative be.

**Fig 4:**
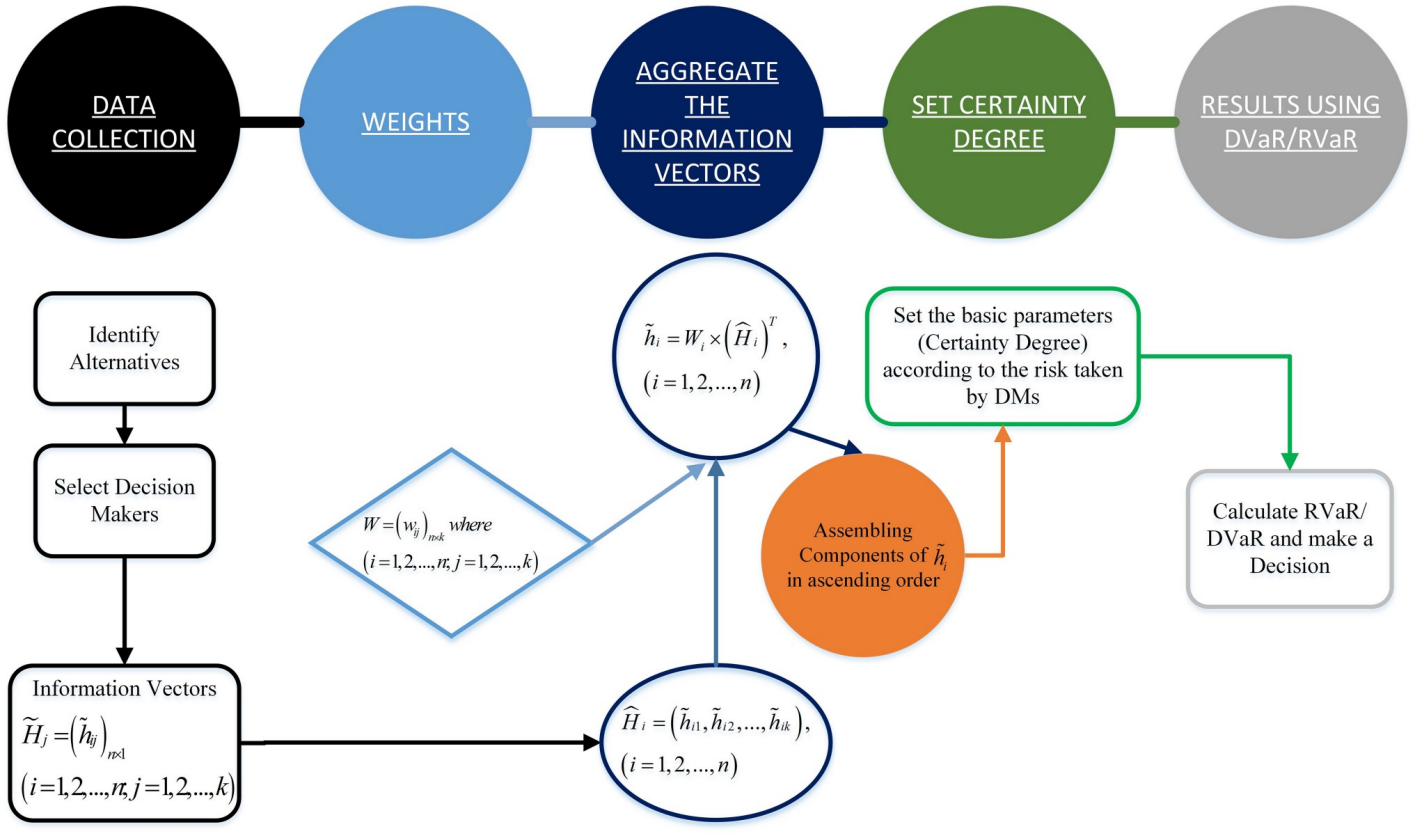
Proposed decision making framework under PIVHF environment.

For the better understanding, complete map of the above proposed model is shown in the Fig 4.

In the next section, we give an example to show the effectiveness of our proposed result TDM under PIVHF environment.

## 6 An illustrative example

Every day, people/companies seek financial opportunities by trading the markets/stock market all over the world. Fund-raising is tough for early-stage companies. An example regarding the stock schemes selection problem from [36] is used to exhibit the execution process of our suggested model:

The three stockholders of a private company want to invest the fund in an international enterprise market (IEM). From the survey of the IEM, stockholders observe that the market experiencing a downward trend, which has begun in July 2019. Therefore, investment in IEM means losing the fund. However, the stockholders observe that the four newly established syndicates listed in 2017, *E*_1_, *E*_2_, *E*_3_ and *E*_4_ are hopeful that the shares will be upswing over a some period of time. So they decided to hold the fund on one of the four stocks and tolerate the loss for some period. For the declined period and suffer a minimum loss, they decide one of the more suitable syndicate to get minimum loss. For the suitable selection, all agree to the following conditions.

1. The main criterion index *C* of the selection is a benefit i.e. anti-risk.
2. The decision focus on the question “how much the worst stock can be.” Thus, our proposed principle is much suitable for selecting the best alternative.
3. As the four companies are newly established, so quantitative information is limited. Therefore, qualitative information is used for selecting the stock provided by the three stockholders and expresses as PIVHFEs.
4. As the three stockholders are equal, but the reliability degrees of the provided PIVHFE information are different. Therefore, the weights can be calculated based on the deviation degree of the three stockholders.

We can see that the TDM approach under PIVHF environment can solve the investment problem. The comprehensive calculation procedure is given below:

**Step 1**. **Collection of PIVHFE information matrices**

For the stocks *E* = {*E*_1_, *E*_2_, *E*_3_, *E*_4_} the three stockholders *i*(*i* = 1, 2, 3) provide the PIVHFE according to the *C* (benefit index). The three PIVHFE vectors $\;{\tilde{H}}_{j}=({\tilde{h}}_{ij})$ (*j* = 1, 2, 3, 4 and *i* = 1, 2, 3) are obtained. The information can be written as PIVHF matrix:

$$\begin{array}{c} {\tilde{H}}_{1}=(\begin{array}{c} \left\{([0.1,0.2],0.3),([0.3,0.4],0.5),([0.6,0.7],0.2)\right\}\\ \left\{([0.2,0.3],0.4),([0.6,0.7],0.6)\right\}\\ \left\{([0.5,0.6],0.3),([0.75,0.85],0.7)\right\}\\ \left\{([0.4,0.5],0.5),([0.5,0.6],0.5)\right\} \end{array}) \end{array}$$

$$\begin{array}{c} {\tilde{H}}_{2}=(\begin{array}{c} \left\{([0.3,0.4],0.5),([0.75,0.85],0.5)\right\}\\ \left\{([0,0.01],0.3),([0.3,0.4],0.6),([0.7,0.8],0.1)\right\}\\ \left\{([0.1,0.2],0.4),([0.3,0.4],0.6)\right\}\\ \left\{([0.2,0.3],0.4),([0.65,0.75],0.6)\right\} \end{array}) \end{array}$$

$$\begin{array}{c} {\tilde{H}}_{3}=(\begin{array}{c} \left\{([0.2,0.3],0.35),([0.7,0.8],0.65)\right\}\\ \left\{([0.2,0.3],0.3),([0.6,0.7],0.5),([0.75,0.85],0.2)\right\}\\ \left\{([0.1,0.2],0.4),([0.3,0.4],0.6)\right\}\\ \left\{([0.25,0.35],0.55),([0.8,0.9],0.45)\right\} \end{array}) \end{array}$$

**Step 2**. **Calculating the weights of the DMs**

1. **Find the Scores of the PIVHFE**
  By using Eq 1, the score of all the PIVHFEs are as follows:
  $$\begin{array}{c} s({\tilde{h}}_{11})=0.23333,s({\tilde{h}}_{21})=0.49,s({\tilde{h}}_{31})=0.725,s({\tilde{h}}_{41})=0.5 \end{array}$$
  $$\begin{array}{c} s({\tilde{h}}_{12})=0.575,s({\tilde{h}}_{22})=0.191,s({\tilde{h}}_{32})=0.27,s({\tilde{h}}_{42})=0.52 \end{array}$$
  $$\begin{array}{c} s({\tilde{h}}_{13})=0.575,s({\tilde{h}}_{23})=0.37333,s({\tilde{h}}_{33})=0.31333,s({\tilde{h}}_{43})=0.5475 \end{array}$$
2. **Deviation degree of all the PIVHFEs**
  To find the weights of the DMs, deviation degree of all the PIVHFEs is required. Deviations degree can be calculated by the Eq 2 as follows:
  $$\begin{array}{c} d({\tilde{h}}_{11})=0.04361,d({\tilde{h}}_{21})=0.03840,d({\tilde{h}}_{31})=0.01313,d({\tilde{h}}_{41})=0.00250 \end{array}$$
  $$\begin{array}{c} d({\tilde{h}}_{12})=0.05063,d({\tilde{h}}_{22})=0.05680,d({\tilde{h}}_{32})=0.00960,d({\tilde{h}}_{42})=0.04860 \end{array}$$
  $$\begin{array}{c} d({\tilde{h}}_{13})=0.05688,d({\tilde{h}}_{23})=0.07924,d({\tilde{h}}_{33})=0.05814,d({\tilde{h}}_{43})=0.07487 \end{array}$$
3. **Weights of DMs**

Eq 5 is used to calculate the dynamic weights of DMs as follows:

*min* $P_{1}=(t_{12}^{+}+t_{12}^{-})+(t_{13}^{+}+t_{13}^{-})+(t_{23}^{+}+t_{23}^{-})$

$$\begin{array}{c} \left\{ \begin{array}{c} 0.04361w_{11}-0.05063w_{12}-t_{12}^{+}+t_{12}^{-}=0\\ 0.04361w_{11}-0.05688w_{13}-t_{13}^{+}+t_{13}^{-}=0\\ 0.05063w_{12}-0.05688w_{13}-t_{22}^{+}+t_{23}^{-}=0\\ w_{11}+w_{12}+w_{13}=1,w_{12}=w_{13}\\ w_{11},w_{12,}w_{13}\geq 0\\ t_{12}^{+},\;t_{12}^{-},\;t_{13}^{+},\;t_{13}^{-},\;t_{23}^{+},\;t_{23}^{-}\geq 0 \end{array}\right. \end{array}$$

*min* $P_{2}=(t_{12}^{+}+t_{12}^{-})+(t_{13}^{+}+t_{13}^{-})+(t_{23}^{+}+t_{23}^{-})$

$$\begin{array}{c} \left\{ \begin{array}{c} 0.03840w_{21}-0.05680w_{22}-t_{12}^{+}+t_{12}^{-}=0\\ 0.03840w_{21}-0.07924w_{23}-t_{13}^{+}+t_{13}^{-}=0\\ 0.05680w_{22}-0.07924w_{23}-t_{22}^{+}+t_{23}^{-}=0\\ w_{21}+w_{22}+w_{23}=1\\ w_{21},w_{22,}w_{23}\geq 0\\ t_{12}^{+},\;t_{12}^{-},\;t_{13}^{+},\;t_{13}^{-},\;t_{23}^{+},\;t_{23}^{-}\geq 0 \end{array}\right. \end{array}$$

*min* $P_{3}=(t_{12}^{+}+t_{12}^{-})+(t_{13}^{+}+t_{13}^{-})+(t_{23}^{+}+t_{23}^{-})$

$$\begin{array}{c} \left\{ \begin{array}{c} 0.01313w_{31}{-}_{0.00960}w_{32}-t_{12}^{+}+t_{12}^{-}=0\\ 0.01313w_{31}-0.05814w_{33}-t_{13}^{+}+t_{13}^{-}=0\\ 0.01313w_{32}-0.05814w_{33}-t_{22}^{+}+t_{23}^{-}=0\\ w_{31}+w_{32}+w_{33}=1\\ w_{31},w_{32,}w_{33}\geq 0\\ t_{12}^{+},\;t_{12}^{-},\;t_{13}^{+},\;t_{13}^{-},\;t_{23}^{+},\;t_{23}^{-}\geq 0 \end{array}\right. \end{array}$$

*min* $P_{4}=(t_{12}^{+}+t_{12}^{-})+(t_{13}^{+}+t_{13}^{-})+(t_{23}^{+}+t_{23}^{-})$

$$\begin{array}{c} \left\{ \begin{array}{c} 0.00250w_{41}-0.04860w_{42}-t_{12}^{+}+t_{12}^{-}=0\\ 0.00250w_{41}-0.07487w_{43}-t_{13}^{+}+t_{13}^{-}=0\\ 0.04860w_{42}-0.07487w_{43}-t_{22}^{+}+t_{23}^{-}=0\\ w_{41}+w_{42}+w_{43}=1\\ w_{41},w_{42,}w_{43}\geq 0\\ t_{12}^{+},\;t_{12}^{-},\;t_{13}^{+},\;t_{13}^{-},\;t_{23}^{+},\;t_{23}^{-}\geq 0 \end{array}\right. \end{array}$$

By solving the above systems of linear equalities, we get the weights

$$\begin{array}{c} W_{1}=(0.3372,0.3711,0.3314) \end{array}$$

$$\begin{array}{c} W_{2}=(0.4628,0.3129,0.2243) \end{array}$$

$$\begin{array}{c} W_{3}=(0.3857,0.5273,0.0871) \end{array}$$

$$\begin{array}{c} W_{4}=(0.9218,0.0474,0.0308) \end{array}$$

weight matrix is *W* = (*w*_*ij*_)_4×3_

$$\begin{array}{c} W=(\begin{array}{ccc} 0.3372 & 0.3711 & 0.3314\\ 0.4628 & 0.3129 & 0.2243\\ 0.3857 & 0.5273 & 0.0871\\ 0.9218 & 0.0474 & 0.0308 \end{array}) \end{array}$$

It is observed that the weights are variable. As weight values are calculated using the deviation degree of the PIVHFEs provided by the DMs, that is, the bigger weight allotted to that DM who gave more authentic data.

**Step 3**. **Aggregate** *k* **PIVHFE information vectors**

From ${\tilde{H}}_{j}(i=1,2,3)$, extract ${\tilde{h}}_{ij}(i=1,2,3,4;j=1,2,3)$ and obtain the four vectors ${\hat{H}}_{i}=({\tilde{h}}_{i1},{\tilde{h}}_{i2},{\tilde{h}}_{i3}),(i=1,2,3,4)$ as follows:

$$\begin{array}{c} {\hat{H}}_{1}=(\begin{array}{c} \{([0.1,0.2],0.3),([0.3,0.4],0.5),([0.6,0.7],0.2)\},\\ \{([0.3,0.4],0.5),([0.75,0.85],0.5)\},\\ \left\{([0.2,0.3],0.35),([0.7,0.8],0.65)\right\} \end{array}) \end{array}$$

$$\begin{array}{c} {\hat{H}}_{2}=(\begin{array}{c} \{([0.2,0.3],0.4),([0.6,0.7],0.6)\},\\ \{([0,0.01],0.3),([0.3,0.4],0.6),([0.7,0.8],0.1)\},\\ \left\{([0.2,0.3],0.3),([0.6,0.7],0.5),([0.75,0.85],0.2)\right\} \end{array}) \end{array}$$

$$\begin{array}{c} {\hat{H}}_{3}=(\begin{array}{c} \{([0.5,0.6],0.3),([0.75,0.85],0.7)\},\\ \{([0.1,0.2],0.4),([0.3,0.4],0.6)\},\\ \left\{([0.1,0.2],0.2),([0.4,0.5],0.4),([0.6,0.7],0.4)\right\} \end{array}) \end{array}$$

$$\begin{array}{c} {\hat{H}}_{4}=(\begin{array}{c} \{([0.4,0.5],0.5),([0.5,0.6],0.5)\},\\ \{([0.2,0.3],0.4),([0.65,0.75],0.6)\},\\ \left\{([0.25,0.35],0.55),([0.8,0.9],0.45)\right\} \end{array}) \end{array}$$

Now multiply the weight vectors to obtain the overall PIVHFE information vector $\tilde{H}=({\tilde{h}}_{i})(i=1,2,3,4)$ as follows:

$$\begin{array}{c} {\tilde{h}}_{1}=\{\begin{array}{c} ([0.214817741,0.318196285],0.0525),([0.432710898,0.549852116],0.0975),\\ ([0.464166165,0.59239932],0.0525),([0.612863522,0.730889434],0.0975),\\ ([0.27861524,0.381228901],0.0875),([0.478804178,0.591468197],0.1625),\\ ([0.507703648,0.63008192],0.0875),([0.644319071,0.755768651],0.1625),\\ ([0.402669716,0.510195572],0.035),([0.568432735,0.676615979],0.065),\\ \left([0.592362445,0.707181681],0.035),([0.705484504,0.806672296],0.065\right) \end{array}\} \end{array}$$

$$\begin{array}{c} {\tilde{h}}_{2}=\{\begin{array}{c} ([0.142146495,0.219808677],0.036),([0.265668595,0.354845215],0.06),\\ ([0.339142246,0.447740882],0.024),([0.23273691,0.332962286],0.072),\\ ([0.34321492,0.448414048],0.12),([0.40892966,0.527836764],0.048),\\ ([0.411421531,0.527002169],0.012),([0.49617079,0.608869254],0.02),\\ ([0.546581506,0.665188067],0.008),([0.377561499,0.472889752],0.054),\\ ([0.467186255,0.564122685],0.09),([0.520497023,0.626884545],0.036),\\ ([0.443291792,0.549338214],0.108),([0.52345206,0.627339347],0.18),\\ ([0.571133143,0.680998656],0.072),([0.572941187,0.68043479],0.018),\\ \left([0.634433273,0.735745556],0.03),([0.671010113,0.773795484],0.012\right) \end{array}\} \end{array}$$

$$\begin{array}{c} {\tilde{h}}_{3} \end{array}=\{\begin{array}{l} ([0.282570338,0.387687639],0.024),([0.307464916,0.412247918],0.048),\\ ([0.33149566,0.43782549],0.048),([0.371612627,0.473870285],0.036),\\ ([0.393417466,0.494973717],0.072),([0.414465684,0.516951259],0.072),\\ ([0.450873934,0.580553199],0.056),([0.469928432,0.597377505],0.112),\\ ([0.488321744,0.614898678],0.112),([0.519027573,0.639590118],0.084),\\ \left([0.535717161,0.654046411],0.168),([0.551827625,0.669101488],0.168\right) \end{array}\}$$

$$\begin{array}{c} {\tilde{h}}_{4} \end{array}=\{\begin{array}{l} ([0.387567577,0.48783968],0.11),([0.411999072,0.516531485],0.09),\\ ([0.411101382,0.512234996],0.165),([0.434594053,0.539560147],0.135),\\ ([0.482311041,0.583059312],0.11),([0.502962977,0.606416805],0.09),\\ \left([0.502204161,0.602919109],0.165),([0.522062509,0.625164033],0.135\right) \end{array}\}$$

**Step 4**. **Set the certainty degree of the PIVHFE**

We use five basic parameters (certainty degrees) 10%, 20%, 30%, 40%, and 50% to calculate the *R*_*i*_. The values against the five certainty degrees are given in the Tables 1 and 2.

**Table 1:** DVaR results under PIVHF environment.

Sr.	Certainty degree	*E*_1_	*E*_2_	*E*_3_	*E*_4_	Classification by proposed DVaR
1	10%	0.329922	0.28285	0.384661	0.437704	*E*_4_ ≺ *E*_3_ ≺ *E*_1_ ≺ *E*_2_
2	20%	0.491282	0.395814	0.444196	0.461668	*E*_1_ ≺ *E*_4_ ≺ *E*_3_ ≺ *E*_2_
3	30%	0.528283	0.395814	0.465708	0.464265	*E*_1_ ≺ *E*_3_ ≺ *E*_4_ ≺ *E*_2_
4	40%	0.535136	0.468383	0.533653	0.487077	*E*_1_ ≺ *E*_3_ ≺ *E*_4_ ≺ *E*_2_
5	50%	0.568893	0.496315	0.55161	0.487077	*E*_1_ ≺ *E*_3_ ≺ *E*_2_ ≺ *E*_4_

**Table 2:** RVaR results under PIVHF environment.

Sr.	certainty degree	*E*_1_	*E*_2_	*E*_3_	*E*_4_	Classification by proposed RVaR
1	10%	0.059326	0.049325	0.072173	0.087541	*E*_4_ ≺ *E*_3_ ≺ *E*_1_ ≺ *E*_2_
2	20%	0.142234	0.11621	0.157086	0.179395	*E*_4_ ≺ *E*_3_ ≺ *E*_1_ ≺ *E*_2_
3	30%	0.242525	0.195373	0.249023	0.271859	*E*_4_ ≺ *E*_3_ ≺ *E*_1_ ≺ *E*_2_
4	40%	0.34921	0.282647	0.353745	0.366308	*E*_4_ ≺ *E*_3_ ≺ *E*_1_ ≺ *E*_2_
5	50%	0.457081	0.38077	0.461625	0.463724	*E*_4_ ≺ *E*_3_ ≺ *E*_1_ ≺ *E*_2_

**Step 5**. **Conclusion by using the RVaR**

By using the Eqs 3 and 4, we obtain the final values against the certainty degrees, as shown in Tables 1 and 2 and provides the complete information for the alternatives as follows:

1. When the DVaR method is used to find the alternative, then following results are obtained:
  (a) If the three distributors focus on the criteria (anti-risk) under 10%, then the best choice is *E*_4_.
  (b) If the three distributors focus on the criteria (anti-risk) under 20%, 30%, 40% or 50%, then the best alternative is *E*_1_ and easily see that *E*_4_ is now on the second and third preferences.
2. When the RVaR method is used to find the alternative, then following results are obtained:

If the three distributors focuses on the criteria (anti-risk) under 10%, 20%, 30%, 40% or 50% then the best alternative is *E*_4_ and worst is *E*_2_.

Thus, the three distributors can select the optimal alternative according to the requirement or certainty degrees under the risk level, which they have to bear. As we see that some differences arise based on DVaR and RVaR measurements. But it is already mentioned in Section 4.1 and 4.2 that RVaR is more suitable instead of DVaR measurement. The two main reasons for measuring the DVaR is

1. To show the effectiveness of the DVaR in TDM under the PIVHF environment.
2. Also, to compare the result with RVaR, which shows that RVaR is better than DVaR and gives us more consistent results for TDM.

## 7 Comparison analysis

In this section, we compare the proposed methodology with the existing approach EVaR to exhibit the validity and advantages of the RVaR method. The data used in the above example is taken by the paper of Zhou and Xu [36] so that we can compare the results. Ranking under different certainty degrees can be seen in Tables 3 and 4 using HVaR and EVaR calculated by Zhou and Xu.

**Table 3:** HVaR results under PHF environment.

Sr.	Certainty degree	Ranking
1	10%	*E*_4_ ≺ *E*_3_ ≺ *E*_1_ ≺ *E*_2_
2	20%	*E*_4_ ≺ *E*_1_ ≺ *E*_3_ ≺ *E*_2_
3	30%	*E*_3_ ≺ *E*_4_ ≺ *E*_1_ ≺ *E*_2_
4	40%	*E*_3_ ≺ *E*_4_ ≺ *E*_1_ ≺ *E*_2_
5	50%	*E*_1_ ≺ *E*_3_ ≺ *E*_4_ ≺ *E*_2_

**Table 4:** EVaR results under PHF environment.

Sr.	Certainty degree	Ranking
1	10%	*E*_4_ ≺ *E*_3_ ≺ *E*_1_ ≺ *E*_2_
2	20%	*E*_4_ ≺ *E*_3_ ≺ *E*_1_ ≺ *E*_2_
3	30%	*E*_4_ ≺ *E*_3_ ≺ *E*_1_ ≺ *E*_2_
4	40%	*E*_3_ ≺ *E*_4_ ≺ *E*_1_ ≺ *E*_2_
5	50%	*E*_3_ ≺ *E*_1_ ≺ *E*_2_ ≺ *E*_4_

The results obtained from the proposed DVaR and the method HVaR defined by Zhou and Xu [36] can be seen in Tables 1 and 3 respectively. If the distributors focus on the criteria (anti-risk) under 10%, then by DVaR the best choice is *E*_4_ and worst is *E*_2_ and the same preferences can be obtained when the HVaR is used. However, If the distributors focus on the criteria (anti-risk) under 20%, 30%, 40% or 50%, then the best alternative is *E*_1_ and we see that *E*_4_ is now on the second and third preferences and the worst is *E*_2_. As these results are carried out through the process of DVaR and we can easily see that these results are not authentic because for 10% *E*_4_ is the best while for the criteria of 20%, 30%, 40% or 50% *E*_1_ is the best alternative. We already mentioned in Section 4 that the results derived from DVaR may be wrong because it uses less information.

The results obtained from the proposed RVaR and the method EVaR defined by Zhou and Xu [36] can be seen in Tables 2 and 4 respectively. If the three distributors focuses on the criteria (anti-risk) under 10%, 20%, 30%, 40% or 50% then the best alternative is *E*_4_ and worst is *E*_2_ using RVaR. However, the results obtained from the EVaR shown in Table 3, under criteria 10%, 20% and 30%, the best alternative is *E*_4_ and worst is *E*_2_ while for the criteria of 40% and 50% the best is *E*_3_ and worst is *E*_2_ and *E*_4_ respectively. From the processed values of *E*_1_, *E*_2_, *E*_3_, and *E*_4_ we see that there is a proportional increase in values with change in criteria percentage. This also shows that RVaR produces better results than DVaR. The RVaR results show consistency when compared with EVaR so our proposed method provides authentic and better results.

## 8 Conclusions and future prospects

This study develops a new decision framework under the context of the probabilistic interval-valued hesitant fuzzy environment, based on tail information and the framework has been applied to the tail group decision making. First, this paper introduced the concepts of the HFS, the PHFS, the PIVHFS, the PIVHFE, and the VaR as the basis of the proposed method to achieve the objective. Then, two new VaRs namely the deemed VaR and the reckoned VaR are introduced along with their operational laws have been explored. The feasibility and effectiveness of the DVaR and RVaR are tested through the examples given in Section 4.1 and 4.2 under the environment of PIVHFS. The proposed operators, such as DVaR and RVaR, are more reliable than HVaR and EVaR operators in diminishing decision information loss. The framework integrates a novel programming model for the weight calculation of DMs to distinguish the PIVHFE information with different accuracy degrees. Furthermore, a new tail group decision-making model based on RVaR under the PIVHF environment has been provided. Finally, we gave a case study on the proposed decision-making method in selecting the optimal stock for four newly listed stocks in the GEM board of the Shenzhen Stock Exchange to validate the efficiency of the proposed methods. The tail information under the PIVHF environment was mainly presented to decision making. The approach mentioned above handled the loss of data in TGDM problems appropriately. The reason is that PIVHFEs can efficiently reduce information loss, thus making the procedure more reliable and consistent. The proposed operators explained the possibility and usefulness using the example of stock selection. To sum up, the following conclusions can be drawn:

1. The decision is taken purely using tail information; therefore, the proposed method (DVaR and RVaR) provide a solution to the question, “How bad/good can a thing be” or in a statement” we are *X* percent certain that the expected value of the aggregated result will not less than *R*. It makes the difference from the traditionally used decision making approaches.
2. The optimal alternatives relying on TDM methods vary depending on the degree of certainty of DVaR or RVaR. But RVaR produces more accurate and specific results, which can be shown in Tables 2 and 3.

It can be concluded that the proposed technique is more flexible, reliable, and general for explaining the interrelationship among the multiple input arguments and offered the space for DMs to display the fuzzy information. We are intended to extend the idea in the future to multi-criteria decision making in a more generalized way that can handle all types of ambiguous information and compare the TDM with the other well-known methodologies.
